# Immunosuppression by hydroxychloroquine: mechanistic proof in in vitro experiments but limited systemic activity in a randomized placebo-controlled clinical pharmacology study

**DOI:** 10.1007/s12026-023-09367-3

**Published:** 2023-02-22

**Authors:** Aliede E. in ‘t Veld, Hendrika W. Grievink, Johan L. van der Plas, Boukje C. Eveleens Maarse, Sebastiaan J. W. van Kraaij, Tess D. Woutman, Mascha Schoonakker, Naomi B. Klarenbeek, Marieke L. de Kam, Ingrid M. C. Kamerling, Manon A. A. Jansen, Matthijs Moerland

**Affiliations:** 1 Centre for Human Drug Research, Leiden, The Netherlands; 2 Leiden University Medical Centre, Leiden, The Netherlands; 3 Division of BioTherapeutics, Leiden Academic Center for Drug Research, Leiden University, Leiden, The Netherlands

**Keywords:** Hydroxychloroquine, Clinical study, Toll-like receptor, T cell, B cell, Autoimmunity

## Abstract

Based on its wide range of immunosuppressive properties, hydroxychloroquine (HCQ) is used for the treatment of several autoimmune diseases. Limited literature is available on the relationship between HCQ concentration and its immunosuppressive effect. To gain insight in this relationship, we performed in vitro experiments in human PBMCs and explored the effect of HCQ on T and B cell proliferation and Toll-like receptor (TLR)3/TLR7/TLR9/RIG-I-induced cytokine production. In a placebo-controlled clinical study, these same endpoints were evaluated in healthy volunteers that were treated with a cumulative dose of 2400 mg HCQ over 5 days. In vitro, HCQ inhibited TLR responses with IC_50_s > 100 ng/mL and reaching 100% inhibition. In the clinical study, maximal HCQ plasma concentrations ranged from 75 to 200 ng/mL. No ex vivo HCQ effects were found on RIG-I-mediated cytokine release, but there was significant suppression of TLR7 responses and mild suppression of TLR3 and TLR9 responses. Moreover, HCQ treatment did not affect B cell and T cell proliferation. These investigations show that HCQ has clear immunosuppressive effects on human PBMCs, but the effective concentrations exceed the circulating HCQ concentrations under conventional clinical use. Of note, based on HCQ’s physicochemical properties, tissue drug concentrations may be higher, potentially resulting in significant local immunosuppression. This trial is registered in the International Clinical Trials Registry Platform (ICTRP) under study number NL8726.

**Supplementary Information:**

The online version contains supplementary material available at 10.1007/s12026-023-09367-3.

## Introduction

Hydroxychloroquine (HCQ) is a broad immunosuppressive drug, initially developed as an antimalarial drug. However, due to its anti-inflammatory properties, HCQ is now widely used in the treatment of autoimmune diseases such as rheumatoid arthritis (RA) [1], systemic lupus erythematosus (SLE) [2], and Sjögren’s syndrome [3]. The use of HCQ in other diseases has been under investigation, a pilot trial investigating the use of HCQ in patients after myocardial infarction showed a decrease in plasma IL-6 levels compared to placebo, and a larger trial studying the effect on recurrent cardiovascular events is currently ongoing [4]. Furthermore, HCQ was under investigation for use in moderate to severe COVID-19 patients during the COVID-19 pandemic [5].

The exact mechanisms behind HCQ immunosuppressive functions remain unclear. HCQ accumulates in the lysosomes and inhibits lysosomal function by autophagosome fusion with lysosomes [6], thereby inhibiting antigen presentation [7, 8]. In addition, HCQ inhibits proinflammatory cytokine production by myeloid cells, possibly via the inhibition of endosomal Toll-like receptor (TLR) signaling [9]. It has been shown that HCQ treatment is associated with decreased interferon (IFN)α serum levels in SLE patients [10]. Furthermore, several studies investigating the effect of HCQ on peripheral blood mononuclear cells (PBMCs) or cell lines show that HCQ treatment reduces phorbol 12-myristate 13-acetate (PMA) and ionomycin or lipopolysaccharide-induced cytokine production [11–13].

Besides effects on the innate immune system, HCQ affects the adaptive immune response as well. It has been shown that HCQ inhibits differentiation of class-switched memory B cells into plasmablasts and thereby decreases IgG production in response to TLR9 stimulation or inoculation with inactivated virus [14, 15]. HCQ inhibits T cell activation as well, via the inhibition of T cell receptor-induced calcium mobilization and dysregulation of mitochondrial superoxide production [16–18].

However, the concentrations used in such in vitro experiments studying the immunomodulatory effects of HCQ largely exceeded obtainable clinical concentrations in patients. A study in cutaneous lupus erythematosus patients receiving HCQ in clinical doses showed that higher HCQ blood levels corresponded with lower ex vivo IFNα responses after TLR9 stimulation, but not after TLR7/8 stimulation [13]. Moreover, influenza antibody titers after vaccination in Sjögren’s syndrome patients receiving HCQ were lower compared to HCQ naïve patients [15]. Unfortunately, little additional literature is available on the in vivo immunomodulatory effects of HCQ and comparing it to in vitro experiments.

We aimed to assess and quantify the immunomodulatory effects of HCQ on primary human immune cells, both in vitro and ex vivo in a randomized clinical trial. We assessed the effect of HCQ on cytokine production after endosomal TLR stimulation in isolated PBMCs and on T and B cell proliferation (in vitro as well as ex vivo). In the clinical trial, healthy subjects were dosed with HCQ in the standard dosing regimen for moderate-to-severe COVID-19 that was advised in the Netherlands when the study was conceived. In the study design, we accounted for a potential age effect on the study outcomes, since general immunocompetence and drug metabolism have been reported to be age-dependent [19, 20]. Here, we present the outcomes of the in vitro experiment and the randomized clinical trial.

## Methods

### In vitro experiments

Blood was collected by venipuncture using sodium heparin vacutainer tubes or Cell Preparation Tubes (CPT, Becton Dickinson, Franklin Lakes, NJ, USA) from healthy volunteers after written informed consent, in accordance with Good Clinical Practice guidelines and the Declaration of Helsinki. Blood was used for the evaluation of the in vitro immunomodulatory activity of hydroxychloroquine (10–10,000 ng/mL, Sigma-Aldrich, Deisenhofen, Germany). All experiments were started within one hour after blood withdrawal, and incubations were performed in duplicate. Hydroxychloroquine and stimulant were added simultaneously. Per experiment, blood of 6 donors was used.

### Clinical study

We conducted a single-blind, randomized, placebo-controlled multiple dose study in forty healthy male volunteers, comprising twenty young (18–30 years) and twenty elderly (65–75 years) subjects. The study was conducted at the Centre for Human Drug Research in Leiden, The Netherlands, between June and September 2020, during the COVID-19 pandemic. All subjects in the clinical trial gave written informed consent according to Declaration of Helsinki recommendations, prior to any study-related activity. The study was approved by the Independent Ethics Committee of the Foundation “Evaluation of Ethics in Biomedical Research” (Stichting Beoordeling Ethiek Biomedisch Onderzoek, Assen, The Netherlands) and registered in the Toetsingonline Registry (study number NL73816.056.20) and in the International Clinical Trials Registry Platform (NL8726).

#### Volunteer selection

To avoid sex-related interindividual variability in immune responses, only male subjects were included [21]. Subjects were included if they were overtly healthy. The health status of subjects was assessed by medical screening, including medical history, physical examination, vital signs measurements, 12-lead electrocardiography (ECG), urine analysis, drug screen and safety chemistry, coagulation, and hematology blood sampling. BMI of study participants had to be between 18 and 32 kg/m^2^. Subjects with a known hypersensitivity reaction to chloroquine, HCQ, or other 4-aminoquinolines, abnormalities in the resting ECG (including QTcF interval > 450 ms), evidence of any active or chronic disease or condition (including long QT syndrome, retinal disease, G6PD deficiency, autoimmune diseases, diabetes mellitus type I or II, and psychiatric disorders), or a positive SARS-CoV-2 PCR test were excluded from study participation. Use of concomitant medication was not permitted during the study and 14 days (or 5 half-lives) prior to the study drug administration, except for paracetamol.

#### Study design

Subjects were randomized to receive either hydroxychloroquine sulfate (Plaquenil®) or placebo tablets, in a 1:1 ratio. Tablets were dispensed by the pharmacy, according to a randomization list generated by a study-independent statistician. Plaquenil® and placebo tablets were packaged in the same way, but the tablets were not indistinguishable; study drug administration was therefore performed by dedicated unblinded personnel not involved in any other study tasks. Subjects received HCQ or placebo by a loading dose of 400 mg twice daily ($t=0 \mathrm{h}$ and $t=12 \mathrm{h}$) followed by a 400 mg once daily dose regimen ($t=24 \mathrm{h}$, $t=48 \mathrm{h}$, $t=72 \mathrm{h}$, and $t=96 \mathrm{h}$), giving a cumulative dose of 2400 mg. This reflected the standard dosing regimen for moderate-to-severe COVID-19 patients in the Netherlands when the study was conceived (total dose between 2000 and 3800 mg).

### Pharmacokinetic evaluation

For pharmacokinetic (PK) assessments, blood was collected in 3 mL Vacutainer® K_2_EDTA tubes (Becton Dickinson) on study day 0 (baseline and 3 h postdosing) and days 1, 4, and 9 (3 h postdosing). Hydroxychloroquine plasma concentrations were measured by Ardena Bioanalytical Laboratory (Assen, the Netherlands) using a validated LC–MS/MS method. The lower limit of quantification (LLOQ) of the analysis was 5 ng/mL.

### Whole blood stimulation

Whole blood was stimulated with 10 μg/mL phytohemagglutinin (PHA, Sigma-Aldrich) for 6 h and 24 h. After 6 h, activation markers on T cells were measured using CD69-APC (clone: REA824), CD71-FITC (clone: REA902), CD154-VioBlue (REA238) and CD25-PE (clone: 3G10), CD3-VioGreen (REA613), CD4-APC-Vio770 (REA623), and CD8-PE-Vio770 (REA734) antibodies and propidium iodide as viability dye (all Miltenyi Biotec, Bergisch-Gladbach, Germany) using a MACSQuant 16 analyzer (Miltenyi Biotec). After 24 h, culture supernatants were collected for cytokine analysis.

### PBMC isolation and TLR stimulation

PBMCs were isolated from CPT after centrifugation at 1800 × *g* for 30 min and washed 2 × using phosphate-buffered saline (PBS, pH 7.2, Gibco, Thermo Fisher, Waltham, MA, USA). PBMCs were stimulated with endosomal TLR ligands poly I:C (TLR3, 50 μg/mL), imiquimod (TLR7, 1 μg/mL), CpG class A (TLR9, oligodeoxynucleotides (ODN) 2.5 μM), and poly I:C/lyovec (RIG-I, 1 μg/mL; all Invivogen, Toulouse, France). Supernatants were collected after 24 h for cytokine quantification.

### Proliferation assay

PBMCs were stained with 2.5 μM cell trace violet (CTV, Thermo Fisher) according to user’s manual. T cells were stimulated with 5 μg/mL phytohemagglutinin (PHA) and B cells with a monoclonal CD40 antibody (5 μg/mL; clone: G28.5, BioXCell) and CpG class B (2.5 μM; ODN Invivogen). After 5 days of stimulation, PBMCs were stained using CD4-PE (clone: OKT4), CD8-APC (clone: HIT8a), CD19-PE (clone: HIB19, all Biolegend, San Diego, CA, USA), and fixable viability dye eFluor780 (Thermo Fisher) and proliferation was quantified by flow cytometry, using the MACSQuant 16 analyzer.

### Flow cytometry

Circulating leukocyte subsets were analyzed using flow cytometry. Red blood cell lysis was performed on sodium heparinized blood using RBC lysis buffer (Thermo Fisher Scientific). After washing with PBS (pH 7.2), leukocytes were incubated with fluorochrome-labeled antibodies for 30 min on ice. After a final washing step, leukocytes were measured on a MACSQuant 16 analyzer (Miltenyi Biotec). See supplemental table I for a full list of antibodies used.

### Cytokine measurements

IFNγ and IL-2 were quantified using the Vplex-2 kit (Meso Scale Discovery). IFNα and IL-6 were quantified using the pan-specific IFNα ELISA^pro^ HRP kit and the IL-6 ELISA^pro^ HRP kit (both Mabtech, Nacka Strand, Sweden).

### Statistical analysis

In vitro data are reported as mean ± standard deviation (SD). The IC_50_ was calculated using an inhibitory sigmoid Emax function where applicable. Analyses were performed using GraphPad Prism version 6.05 (GraphPad, San Diego, CA, USA).

Repeatedly measured pharmacodynamic data were evaluated with a mixed model analysis of variance with fixed factors treatment, age group, time, treatment by time, age group by time, treatment by age group, and treatment by age group by time and a random factor subject and the average prevalue as covariate. If needed, variables were log transformed before analysis. Contrasts between the placebo and HCQ treatment groups were calculated per endpoint. In addition, a potential age-specific HCQ effect was evaluated by comparing the 18–30 years with the 65–75 years age group. For the contrasts, an estimate of the difference (back-transformed in percentage for log-transformed parameters), a 95% confidence interval (in percentage for log-transformed parameters), least square means (geometric means for log transformed parameters), and the $p$ value were calculated. A $p$ value ≤ 0.05 was considered to be statistically significant. All calculations were performed using SAS for Windows V9.4 (SAS Institute, Inc., Cary, NC, USA).

## Results

### Hydroxychloroquine suppressed endosomal TLR-induced IFNα and IL-6 release in vitro

**Fig. 1:**
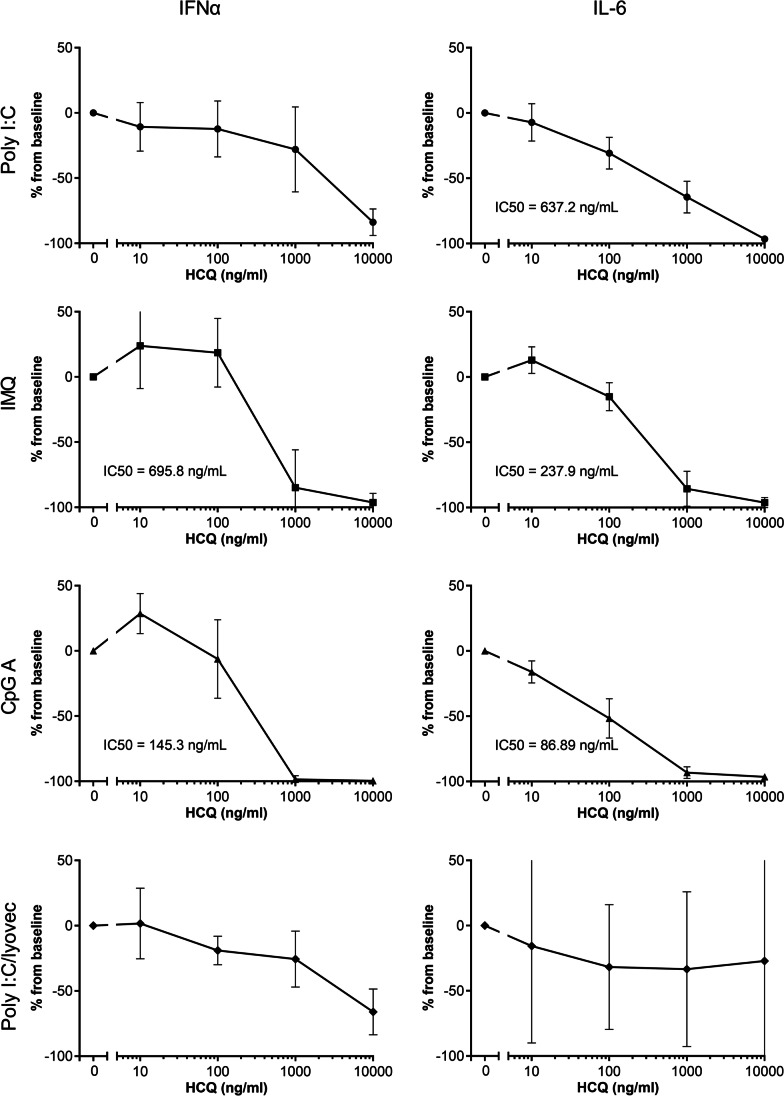
HCQ dose-dependently inhibited endosomal TLR-induced IFNα and IL-6 release in vitro. PBMCs were stimulated with 50 μg/mL poly I:C (TLR3), 1 μg/mL IMQ (TLR7), 2.5 μM CpG-A (TLR9), or 1 μg/mL poly I:C/lyovec (RIG-I) for 24 h in the presence of a dose range of HCQ. IFNα and IL-6 release was measured by ELISA. The mean ± SD of the change from baseline of 6 subjects is shown. The IC_50_ was calculated using a four-parameter nonlinear regression fit where applicable

PBMCs were stimulated with endosomal TLR ligands in the presence of a dose range of HCQ for 24 h, and supernatants were analyzed for IRF-mediated IFNα and for NFκB-mediated IL-6 secretion. PBMCs were stimulated with different endosomal TLR ligands: poly I:C (TLR3), imiquimod (TLR7), CpG class A (TLR9), and poly I:C lyovec (RIG-I). HCQ dose-dependently inhibited endosomal TLR-induced IFNα and IL-6 secretion (Fig. 1). Poly I:C-induced IFNα and IL-6 release was strongly suppressed at 10.000 ng/mL (IFNα: − 83.9%, IL-6: − 96.6%, IC_50_ IL-6 = 637.2 ng/mL). Imiquimod (IMQ)-induced cytokine release was completely suppressed at the highest concentration (IFNα: − 96.3%, IL-6: − 96.3%, IC_50_ IFNα: 695.8 ng/mL, IL-6: 237.9 ng/mL). The same was observed for stimulation with CpG class A, IFNα was suppressed by 99.6% with an IC_50_ of 145.3 ng/mL, and IL-6 was suppressed by 96.4%, with an IC_50_ of 86.9 ng/mL. The RIG-I response to poly I:C/lyovec was less affected by HCQ, while IFNα release was suppressed by 66.1% at 10,000 ng/mL HCQ; IL-6 release was not significantly altered.

### HCQ inhibited B cell proliferation but not T cell proliferation in vitro

**Fig. 2:**
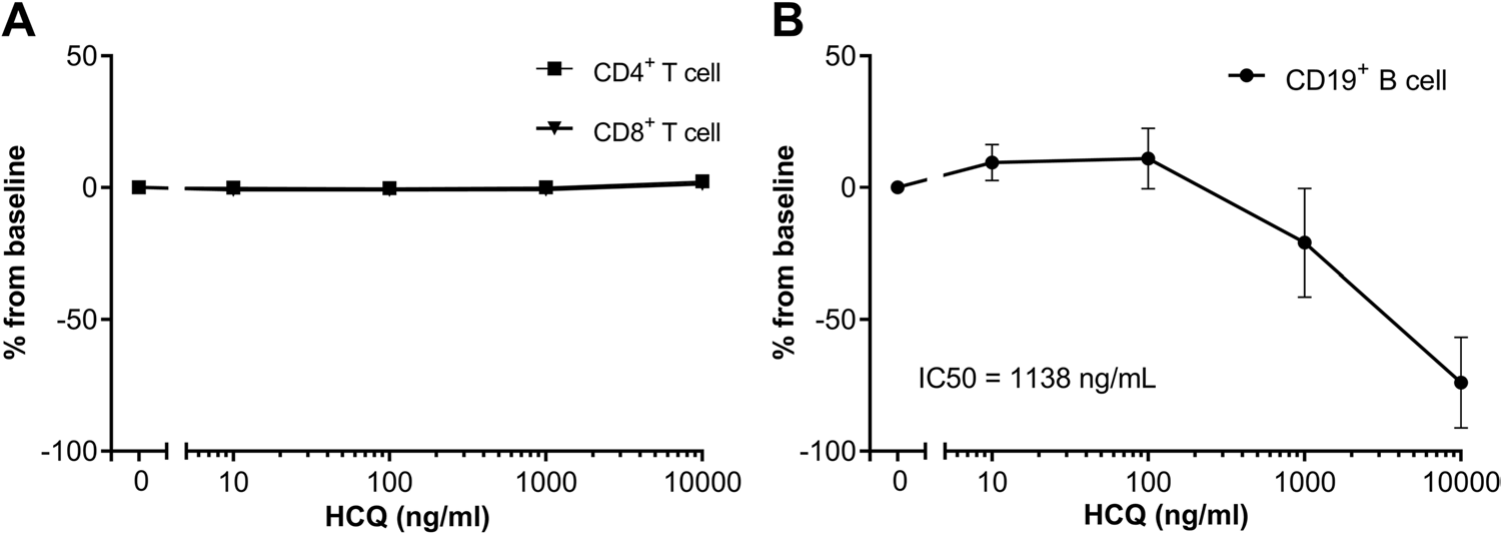
HCQ dose-dependently inhibited B cell but not T cell proliferation in vitro. PBMCs from 6 healthy donors were stained with CTV and stimulated for 5 days with 5 μg/ml PHA for T cell proliferation (**A**) or 5 μg/mL anti-CD40 mAb + 2.5 μM CpG B for B cell proliferation (**B**). Proliferation was measured by flow cytometry. The mean ± SD of the change from baseline are shown. The IC_50_ was calculated using a four-parameter nonlinear regression fit where applicable

PBMCs were stimulated with phytohemagglutinin (PHA) or monoclonal anti-CD40 with CpG-B to induce T cell and B cell proliferation, respectively, in the presence of a dose range of HCQ. No effect of HCQ was seen on T cell proliferation (Fig. 2A). Also, no effects were observed on T cell activation markers after PHA stimulation for 6 h (Figure S1). At HCQ concentrations > 100 ng/mL, a decrease in B cell proliferation was observed, with an IC_50_ of 1138 ng/mL (Fig. 2B).

### Clinical study

#### Demographics and safety

**Fig. 3:**
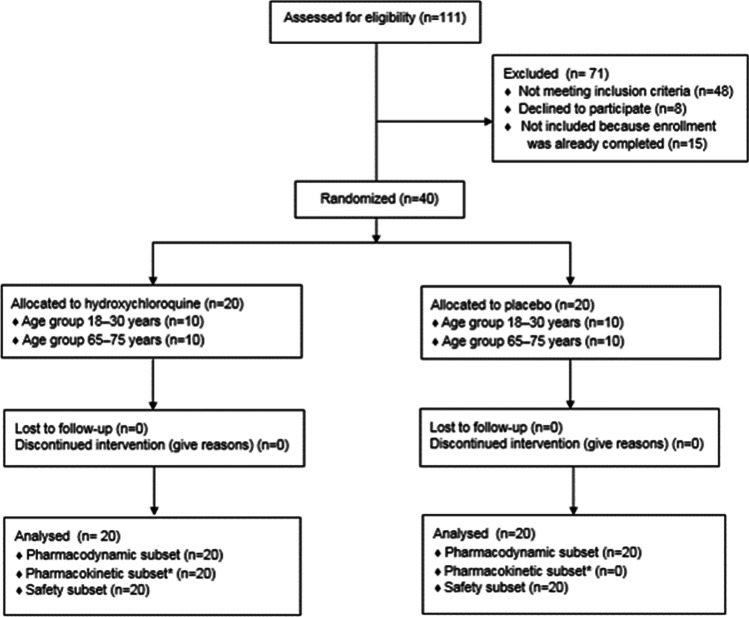
Trial flowchart (CONSORT diagram). *Drug concentrations were only analyzed in the active treatment group

Of the 40 enrolled and randomized healthy subjects, 20 received a cumulative dose of 2400 mg HCQ in 5 days and 20 received placebo (Fig. 3). The different age groups (18–30 and 65–75 years) were of equal size. Baseline characteristics are described in Table 1. All subjects completed their study treatment. One subject in the 65–75 years group erroneously took an additional 400 mg dose of HCQ on study day 2, after which the subject received 400 mg doses (once daily) for two consecutive days to not exceed the cumulative dose of 2400 mg.

**Table 1:** Baseline characteristics

	Hydroxychloroquine		Placebo	
	Age group 18–30 yrs ($n=10$)	Age group 65–75 yrs ($n=10$)	Age group 18–30 yrs ($n=10$)	Age group 65–75 yrs ($n=10$)
Age, median (range)	23 (20–26)	68 (65–70)	23 (18–25)	68 (65–71)
BMI, mean (SD)	21.8 (1.5)	25.8 (2.0)	24.4 (1.9)	24.2 (3.0)
Race or ethnicity*,$n (\%)$				
White Other	10 (100) 0 (0)	10 (100) 0 (0)	10 (100) 0 (0)	10 (100) 0 (0)

^*^Self-reported race or ethnicity of subjects

*BMI* body mass index, *SD* standard deviation

Treatment-emergent adverse events were transient of mild severity and did not lead to study discontinuation. Adverse events were reported more often by subjects in the active treatment arm (50%) compared to placebo (35%). Gastrointestinal complaints (20%) and dizziness (15%) were the most frequently reported adverse events in the active group. There were no findings of clinical concern following assessments of urinalysis, hematology and chemistry laboratory tests, vital signs, physical examination, and ECGs [22].

#### Pharmacokinetics

**Fig. 4:**
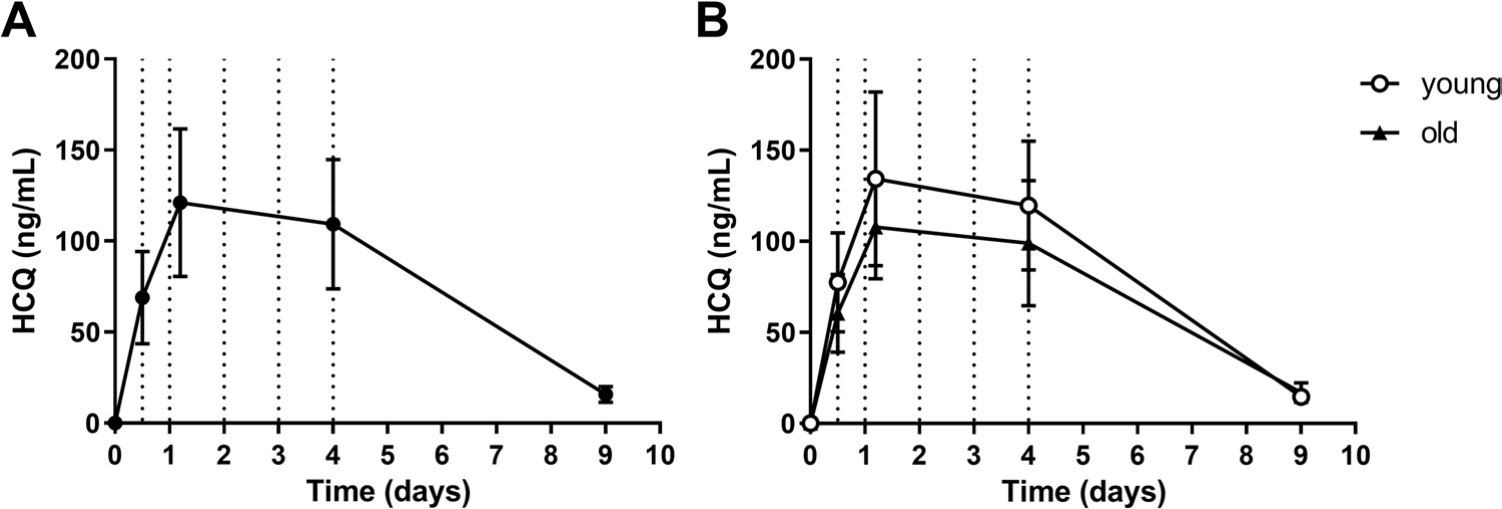
Pharmacokinetic profile of HCQ. Mean and standard deviation of hydroxychloroquine plasma concentrations for HCQ treatment group (**A**) and split for young and elderly volunteers (**B**). Dotted vertical lines indicate timing of HCQ dosing (0, 12, 24, 48, 72, and 96 h)

Mean HCQ concentration time profiles in plasma are depicted in Fig. 4A. Individual concentration profiles have been published previously [22]. There were no significant differences in HCQ exposures between age groups (Fig. 4B). Mean concentrations measured 27 h after starting the treatment course (day 1, 121.0 ± 40.54 ng/mL) were in a similar range to those measured on the last day of the treatment course (day 4, 109.2 ± 35.59 ng/mL).

### Pharmacodynamics

#### Hydroxychloroquine did not affect circulating immune cells

The effects of HCQ on different circulating cell populations, both absolute as relative, were evaluated using flow cytometry. No apparent effects were seen on absolute values of total leukocytes, lymphocytes, monocytes, or neutrophils (Table S2), as well as CD14^+^ monocytes, CD19^+^ B cells, CD3^+^ T cells, CD4^+^ T cells, and CD8^+^ T cells (Table S3). Furthermore, no effects were seen on relative T cell populations (CD3^+^) in general, nor on subpopulations of T helper cells (CD4 +), cytotoxic T cells (CD8^+^), and regulatory T cells (CD4^+^CD25^+^CD127^−^). Similarly, no apparent treatment effects were observed in natural killer cells (CD56^+^), B cells (CD19^+^), and subpopulations of regulatory (CD5^+^CD1d^hi^), transitional (CD24^hi^CD38^hi^), and antibody-secreting B cells (CD27^+^CD38^+^). Moreover, also in classical (CD14^+^), nonclassical (CD16^+^), and intermediate (CD14^+^CD16^+^) monocytes and plasmacytoid dendritic cells (pDCs, HLA-DR^+^CD14^−^CD16^−^CD123^+^), no differences were found between treatment groups. Also, between both age groups, no evident HCQ effects were observed (Table S3).

#### In vivo hydroxychloroquine suppressed IFNα secretion following TLR7 stimulation, but not after TLR3, TLR9, or RIG-I-like receptor stimulation

**Fig. 5:**
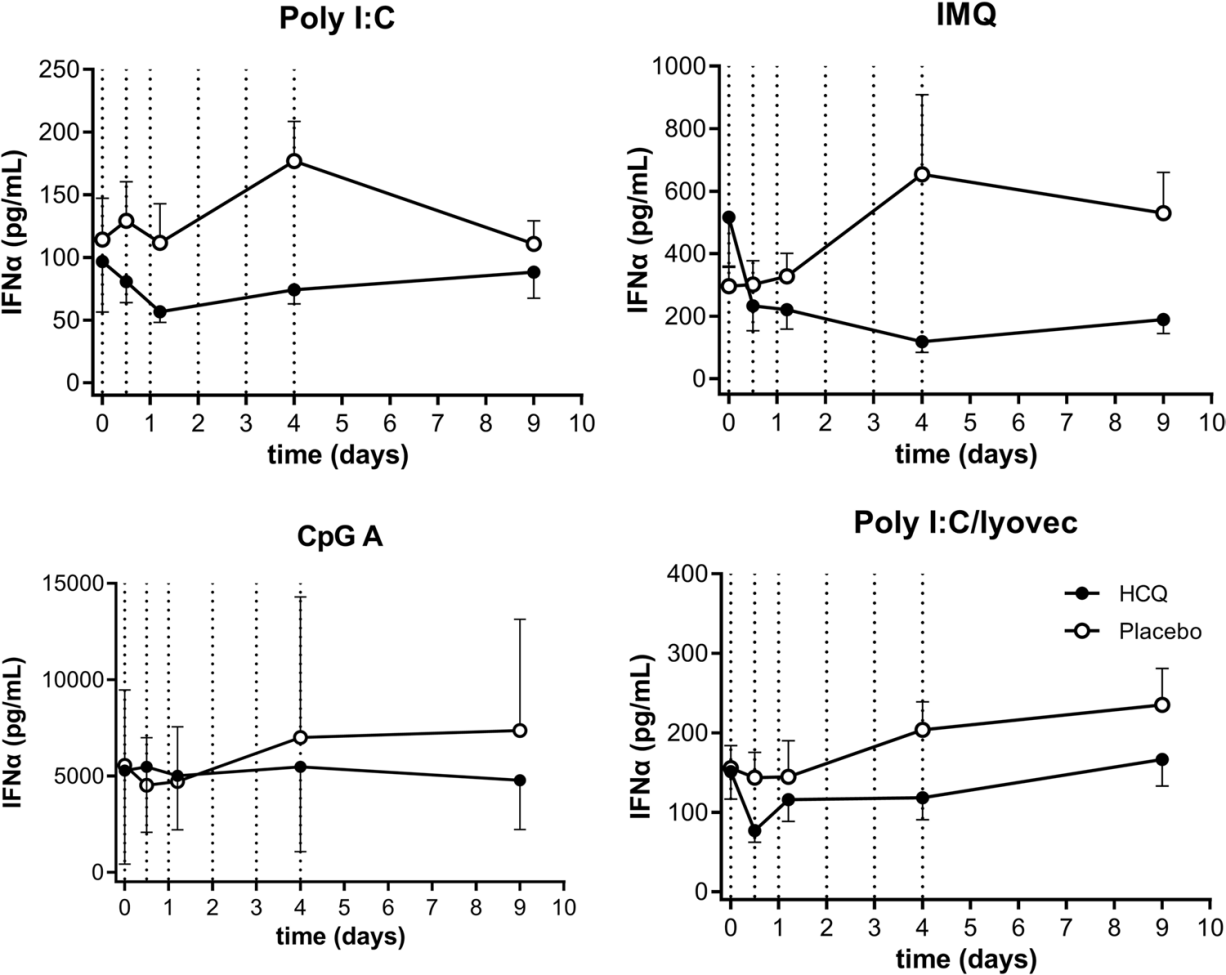
In vivo HCQ inhibited IMQ-induced IFNα release, but not TLR3, TLR9, and RIG-I. PBMCs were stimulated with 50 μg/mL poly I:C (TLR3), 1 μg/mL IMQ (TLR7), 2.5 μM CpG A (TLR9), or 1 μg/mL poly I:C/lyovec (RIG-I) at 0, 12, 24, 48, 72, and 92 h after primary HCQ dosing. IFNα release was measured by ELISA. Data is shown as mean + SD as one-sided error bars. Dotted vertical lines indicate HCQ dosing times

To study the effects of HCQ on TLR/RIG-I-mediated IRF activation, PBMCs were stimulated with different endosomal TLR ligands: poly I:C (TLR3), imiquimod (TLR7), CpG class A (TLR9), and poly I:C lyovec (RIG-I). Overall, no HCQ effect was observed on IFNα responses (Fig. 5), except for a significant suppression of IMQ-driven IFNα production (inhibition of − 48.2%, 95% CI − 72.1%– − 4.0%, *p* = 0.038). Poly I:C-driven IFNα release also appeared to be suppressed by HCQ, but not significantly (inhibition − 34.2%, 95% CI − 57.7%–7.5%, *p* = 0.091). No differences in HCQ effect on IFNα responses were observed between the young and elderly population (Figure S3).

#### In vivo hydroxychloroquine significantly suppressed IL-6 secretion after TLR7 stimulation, but not following TLR3, TLR9, or RIG-I-like receptor stimulation

**Fig. 6:**
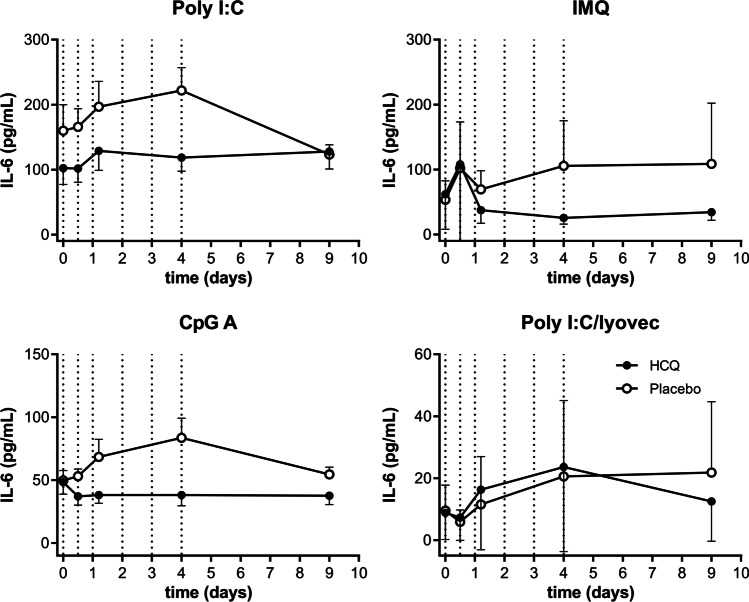
In vivo HCQ inhibited IMQ-induced IL-6 release, but not TLR3, TLR9, and RIG-I. PBMCs were stimulated with 50 μg/mL poly I:C (TLR3), 1 μg/mL IMQ (TLR7), 2.5 μM CpG A (TLR9), or 1 μg/mL poly I:C/lyovec (RIG-I) at 0, 12, 24, 48, 72, and 92 h after primary HCQ dosing. IFNα release was measured by ELISA. Data is shown as mean + SD as one-sided error bars. Dotted vertical lines indicate HCQ dosing times

Activation of NFκB signaling via endosomal TLR and RIG-I-like ligands was assessed by measuring downstream IL-6 production (Fig. 6). HCQ significantly suppressed IMQ-driven IL-6 production (inhibition of − 71.3%, 95% CI − 84.7%– − 46.1%, *p* = 0.0005). No significant HCQ effects were observed on IL-6 production driven by CpG A (TLR9) and poly I:C (TLR3) stimulations (inhibition of − 35.9%, 95% CI − 60. 3%–3.6%, *p* = 0.068, and − 37.7%, 95% CI − 62.6%–3.7%, *p* = 0.067, respectively). No differences in HCQ effect on IL-6 responses were observed between the young and elderly population (Figure S3).

#### In vivo hydroxychloroquine did not alter T cell activation

To further investigate the potential immunomodulatory effect of HCQ on T cell activation*,* whole blood samples were incubated with PHA, which is known to induce a general T cell response [23]. HCQ treatment did not modulate expression of T cell activation markers (CD25, CD69, CD71, and CD154) following PHA stimulation (Figure S3). In addition, PHA-induced secretion of IL-2 and IFNγ was assessed; no apparent differences were observed between HCQ and placebo (Figure S4).

#### Hydroxychloroquine did not alter ex vivo B and T cell proliferation after in vivo administration

**Fig. 7:**
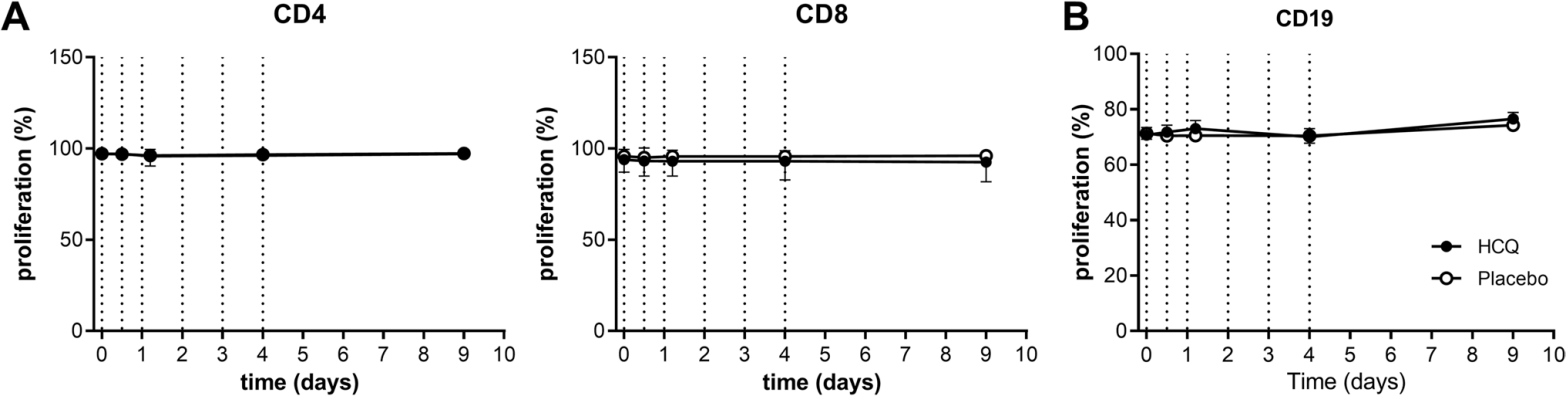
In vivo HCQ did not affect T and B cell proliferation. PBMCs were stained with CTV and stimulated for 5 days with 5 μg/mL PHA for T cell proliferation (**A**) or 5 μg/mL anti-CD40 mAb + 2.5 μM CpG B for B cell proliferation (**B**). Proliferation was measured by flow cytometry. The mean ± SD are shown. Dotted vertical lines indicate HCQ dosing times

Proliferative capability of B cells was assessed by stimulating PBMCs ex vivo with anti-CD40 mAbs + CpG B ODNs, a known stimulus for human B cell activation [24]. Following stimulation of PBMCs, the percentage of proliferative B cells in the HCQ-treated group was similar to that of the placebo group (70.47% at day 4 for placebo, 70.03% for HCQ) (Fig. 7). In addition, PBMCs were stimulated with PHA to induce T helper cell (CD4^+^) and cytotoxic T cell (CD8^+^) proliferation. Proliferation of both CD4^+^ and CD8^+^ cells was comparable between the HCQ- and placebo-treated group (> 95% for both groups for all time points for CD4, > 92% for both groups for all time points for CD8). No differences were observed for B and T cell proliferation in the separate age groups (Figure S5).

## Discussion

Although HCQ is widely used for the treatment of autoimmune diseases, the exact mechanism behind its immunomodulatory properties remains unclear. In this study, we therefore aimed to quantify the immunosuppressive effect of HCQ by studying the endosomal TLR response and lymphocyte proliferation and activation both in in vitro experiments and in vivo in a randomized placebo-controlled trial in healthy volunteers.

In our in vitro experiments, HCQ dose-dependently inhibited TLR3-, 7-, and 9-driven IL-6 and IFNα production, with profound effects at concentrations > 100 ng/mL. These findings are in line with literature on TLR signaling modulation by chloroquine [9, 25]. Limited data are available on the immunomodulatory effect of HCQ/chloroquine on RIG-I signaling [26]. RIG-I functions as a cytosolic sensor of nucleic acids, inducing a type I IFN response after activation. HCQ inhibited the IFN responses in THP-1 cells transfected with RIG-I ligands [27], but this effect was not confirmed in cultures of human bronchial smooth muscle and epithelial cells [28, 29]. This is in line with the observations in the current study, which shows that HCQ only mildly modulated RIG-I-mediated IFNα production in PBMCs, without affecting IL-6 release. Our results suggest that HCQ has a profound effect on endo-lysosomal TLR functioning in vitro but affects the cytosolic RIG-I-mediated pathway to a lesser degree. This could be explained by HCQ’s excessive affinity to the lysosomal intracellular compartment (expected to be 56,000-fold higher than cytosol) [30].

HCQ did not affect T cell activation in vitro. Although a dose-dependent inhibition of T cell proliferation by chloroquine following stimulation with anti-CD3/CD28 has been described [31–33], we did not see any inhibitory effect of HCQ on T cell proliferation or expression of activation markers in our in vitro experiments. This may be explained by the fact that a different and more potent stimulus was used in this study (PHA), which might be more difficult to suppress. For B cell proliferation, on the other hand, a dose-dependent HCQ-mediated inhibition was observed in vitro, confirming previous research [34]*.* Although the HCQ-mediated inhibition was not as strong as the inhibition of cytokine production (IC_50_ of 1138 ng/mL for B cell proliferation vs. 145–696 ng/mL for cytokine production), at concentrations > 100 ng/mL, a clear HCQ-mediated decrease in B cell proliferation was found.

While HCQ had strong immunosuppressive effects in vitro, especially at high concentrations, less pronounced ex vivo effects of the compound were observed in our clinical study. Compared to placebo, 5-day HCQ treatment did not significantly suppress B cell proliferation or ex vivo TLR-driven IFNα and IL-6 secretion in PBMC cultures, except for a suppressive effect on TLR7-driven responses. The most likely explanation for this discrepancy between in vitro and ex vivo is that there was insufficient drug exposure at the evaluated HCQ dose and regimen in the clinical study. By using a 5-day dose regimen of HCQ (the recommended off-label dose for COVID-19 at the time of study conduct), an average maximum plasma concentration of 121 ng/mL was reached. This concentration is considerably lower than plasma levels found in RA patients receiving HCQ treatment of 200 mg daily for a longer time period, which ranges from 200 to 500 ng/mL [35–37]. Peak exposures of 100–150 ng/mL from the clinical study translate into a maximal inhibitory effect of 20 to 50% in most cellular assays. In combination with the observed variability of the endpoints, such effects remain easily undetected. However, whole blood concentrations are expected to be approximately 2-to-sevenfold higher than plasma concentrations due to intracellular uptake in blood components [38–40], which would make the concentrations more in range with the in vitro experiments. Also, due to the large volume of distribution [39] and the high HCQ tissue concentrations as compared to plasma [41, 42], immunosuppressive effects in specific tissues may be significant. Moreover, HCQ has a gradual onset of action for HCQ and is biologically active even after drug discontinuation [8]. This would mean that the five-day treatment that was used in the current study is insufficient to detect ex vivo drug effects. Other studies, for example, investigating HCQ effect in HIV patients [43], showed a discrepancy between plasma levels and drug efficacy.

The widespread use of hydroxychloroquine following the onset of the COVID-19 pandemic was the reason to initiate our experiments. The initial off-label use of HCQ was primarily based on studies that assessed in vitro antiviral activity against SARS-CoV-2 [44]. However, there is also a longstanding hypothesis that the immunomodulatory properties of chloroquine and HCQ could dampen immunopathology caused by viral infections such as influenza, Severe Acute Respiratory Syndrome (SARS), Middle East Respiratory Syndrome (MERS), and COVID-19 by suppressing the host immune response [45–47]. Use of HCQ in COVID-19 patients did not show evident favorable effects for clinical endpoints such as mortality and mechanical ventilation for both prophylaxis and treatment [48]. Our study provides mechanistic insight in the immunomodulatory effects of a HCQ dosing regimen that was used to treat COVID-19. We found that a 5-day treatment course of HCQ did not have extensive immunomodulatory effect in healthy individuals. HCQ treatment only significantly inhibited TLR7 responses. In theory, inhibition of the TLR7-mediated innate response to viral agents may be disadvantageous during the initial stages of viral infection [49, 50]. However, recent COVID-19 trials did not show an effect of HCQ treatment on disease incidence, and long-term HCQ use in rheumatoid arthritis is not associated with higher incidence of upper respiratory tract infections [51, 52].

In conclusion, we showed extensive and profound immunomodulation by HCQ in vitro; however, in a clinical study in healthy volunteers, the overall immunomodulatory effects of a 5-day HCQ treatment regimen of 2400 mg were limited. The pharmacological activity of HCQ in autoimmunity remains to be studied in greater detail, based on the assays as presented in our studies and at a therapeutic dose and regimen relevant for the condition of interest.

## Supplementary Information

Below is the link to the electronic supplementary material.

Supplementary file1 (PDF 487 KB)

## Data Availability

The datasets generated during and/or analyzed during the current study are available from the corresponding author on reasonable request.
