# Supplementary material for: Immunosuppression by hydroxychloroquine: mechanistic proof in in vitro experiments but limited systemic activity in a randomized placebo-controlled clinical pharmacology study
Source: Immunol Res. 2023 Feb 22;71(4):617–27. doi: 10.1007/s12026-023-09367-3 (PMC9945836; doi:10.1007/s12026-023-09367-3)
Supplement: Supplementary file 1 — Supplementary file1 (PDF 487 KB) [file 12026_2023_9367_MOESM1_ESM.pdf]

Table S1: antibodies used for flow cytometry analysis

marker	fluorochrome	clone	supplier
CD45	VioGreen	REA747	Miltenyi Biotec
CD3	AF700	OKT3	Biolegend
CD4	PE-Vio770	REA623	Miltenyi Biotec
CD8	BV570	RPA-T8	Biolegend
HLA-DR	BV605	L243	Miltenyi Biotec
CD19	PerCP-Vio700	REA675	Miltenyi Biotec
CD14	VioBlue	REA599	Miltenyi Biotec
CD16	BV650	3G8	Biolegend
CD123	APC-Vio770	REA918	Miltenyi Biotec
CD56	PE	REA614	Miltenyi Biotec
CD25	VioBright515	REA945	Miltenyi Biotec
CD127	APC	REA614	Miltenyi Biotec
CD19	PerCP-Vio700	REA675	Miltenyi Biotec
CD27	VioBright515	REA499	Miltenyi Biotec
CD38	PE	REA671	Miltenyi Biotec
CD24	VioBlue	REA832	Miltenyi Biotec
CD5	APC-Vio770	REA782	Miltenyi Biotec
CD1d	APC	51,1	Miltenyi Biotec

BV = Brilliant Violet, AF = Alexa Fluor, PE = phycoerythrin, APC = allophycocyanin.

Table S2: circulating immune cells, absolute values, measured by Sysmex

Cell type	Baseline (mean ± SD)	27h (mean ± SD)	4d (mean ± SD)	9d (mean ± SD)
Leukocytes (*10^9/L)				
Young placebo	5.624 ± 0.958	5.487 ± 0.828	4.837 ± 0.919	4.849 ± 0.982
Old placebo	5.749 ± 1.667	6.001 ± 1.931	5.674 ± 1.727	5.730 ± 1.903
Young HCQ	5.218 ± 1.330	5.150 ± 1.118	4.847 ± 1.099	4.731 ± 0.991
Old HCQ	5.363 ± 1.105	5.531 ± 1.277	5.249 ± 0.968	5.090 ± 1.076
Lymphocytes (*10^9/L)				
Young placebo	1.940 ± 0.312	2.101 ± 0.284	1.833 ± 0.306	1.618 ± 0.317
Old placebo	1.647 ± 0.596	1.952 ± 0.843	1.663 ± 0.668	1.689 ± 0.621
Young HCQ	1.720 ± 0.254	1.844 ± 0.299	1.751 ± 0.286	1.568 ± 0.200
Old HCQ	1.474 ± 0.236	1.578 ± 0.323	1.568 ± 0.221	1.403 ± 0.275
Monocytes (*10^9/L)				
Young placebo	0.515 ± 0.068	0.441 ± 0.064	0.438 ± 0.090	0.424 ± 0.101
Old placebo	0.618 ± 0.184	0.546 ± 0.208	0.548 ± 0.122	0.518 ± 0.105
Young HCQ	0.481 ± 0.147	0.412 ± 0.124	0.439 ± 0.120	0.416 ± 0.097
Old HCQ	0.481 ± 0.139	0.433 ± 0.124	0.425 ± 0.081	0.409 ± 0.102
Neutrophils (*10^9/L)				
Young placebo	3.019 ± 0.921	2.823 ± 0.592	2.422 ± 0.710	2.670 ± 0.828
Old placebo	3.257 ± 0.943	3.281 ± 0.936	3.247 ± 1.043	3.299 ± 1.379
Young HCQ	2.794 ± 1.035	2.696 ± 0.811	2.434 ± 0.874	2.504 ± 0.821
Old HCQ	3.262 ± 0.975	3.389 ± 1.028	3.097 ± 0.850	3.137 ± 0.862

Table S3: circulating immune cells, absolute values, measured by flow cytometry

Cell type	Baseline (mean ± SD)	3h (mean ± SD)	27h (mean ± SD)	4d (mean ± SD)	9d (mean ± SD)
$CD14^+$ monocytes (*10^6/L)					
Young placebo	324.9 ± 65.4	349.3 ± 86.8	341.4 ± 58.7	342.9 ± 61.1	375.0 ± 101.2
Old placebo	403.2 ± 138.0	470.7 ± 133.7	450.9 ± 209.8	479.5 ± 87.1	478.0 ± 119.5
Young HCQ	289.2 ± 108.7	328.7 ± 105.1	321.9 ± 120.6	335.8 ± 113.8	343.9 ± 84.9
Old HCQ	314.6 ± 117.7	371.8 ± 93.1	386.3 ± 111.0	363.4 ± 100.3	365.9 ± 129.0
$CD19^+$ B cells (*10^6/L)					
Young placebo	220.4 ± 68.3	264.6 ± 105.5	294.9 ± 93.0	249.8 ± 91.4	217.5 ± 73.0
Old placebo	173.3 ± 102.5	193.8 ± 108.2	191.1 ± 87.6	207.0 ± 102.8	204.5 ± 91.2
Young HCQ	175.1 ± 103.4	211.2 ± 93.0	224.0 ± 106.5	219.1 ± 105.4	197.8 ± 90.2
Old HCQ	191.2 ± 66.6	192.8 ± 72.9	224.6 ± 112.4	205.6 ± 85.2	208.1 ± 86.3
$CD3^+$ T cells (*10^6/L)					
Young placebo	1280 ± 261	1384 ± 377	1499 ± 261	1308 ± 212	1220 ± 246
Old placebo	1296 ± 565	1370 ± 597	1446 ± 726	1345 ± 633	1424 ± 666
Young HCQ	1186 ± 279	1204 ± 226	1373 ± 305	1267 ± 233	1215 ± 215
Old HCQ	1081 ± 241	1158 ± 228	1233 ± 319	1181 ± 233	1115 ± 194
$CD4^+$ T cells (*10^6/L)					
Young placebo	711.1 ± 216.7	733.8 ± 230.7	815.1 ± 187.1	706.0 ± 146.5	672.7 ± 148.5
Old placebo	864.9 ± 367.3	886.2 ± 363.5	951.4 ± 413.4	883.1 ± 375.5	944.1 ± 457.8
Young HCQ	688.2 ± 215.3	674.5 ± 183.4	763.1 ± 202.0	701.2 ± 179.4	687.0 ± 169.2
Old HCQ	756.9 ± 195.9	788.5 ± 185.9	865.0 ± 295.2	806.8 ± 150.1	766.4 ± 160.8
$CD8^+$ T cells (*10^6/L)					
Young placebo	487.2 ± 93.1	551.9 ± 162.8	577.7 ± 106.8	507.5 ± 98.7	463.1 ± 123.6
Old placebo	402.9 ± 271.9	447.0 ± 302.0	453.4 ± 367.4	433.5 ± 300.3	444.6 ± 301.1
Young HCQ	424.6 ± 93.3	454.3 ± 98.6	517.1 ± 151.4	486.2 ± 113.1	446.3 ± 78.7
Old HCQ	286.9 ± 106.4	331.5 ± 143.9	328.2 ± 107.5	335.5 ± 178.3	315.1 ± 153.8

Table S4: circulating immune cells, relative values, measured with flow cytometry

Cell type	Baseline (mean ± SD)	3h (mean ± SD)	27h (mean ± SD)	4d (mean ± SD)	9d (mean ± SD)
CD3 (% of CD45)					
Young placebo	42.77 ± 11.74	42.08 ± 10.28	45.33 ± 12.10	41.72 ± 14.22	45.04 ± 14.44
Old placebo	59.65 ± 7.50	55.73 ± 7.56	58.95 ± 7.35	59.07 ± 9.01	58.67 ± 10.21
Young HCQ	40.03 ± 9.64	41.67 ± 13.36	37.72 ± 12.77	38.63 ± 9.85	42.37 ± 8.51
Old HCQ	61.09 ± 8.33	59.68 ± 7.61	59.62 ± 7.31	62.27 ± 6.29	59.18 ± 7.66
CD4 (% of CD45)					
Young placebo	23.21 ± 8.34	22.20 ± 7.13	25.20 ± 8.08	22.52 ± 9.55	24.85 ± 8.94
Old placebo	40.35 ± 7.30	36.25 ± 6.29	39.39 ± 5.82	39.24 ± 7.97	39.02 ± 8.09
Young HCQ	22.85 ± 7.84	22.35 ± 8.38	21.31 ± 9.26	21.26 ± 7.74	23.84 ± 7.12
Old HCQ	42.71 ± 7.30	41.05 ± 6.27	41.14 ± 6.57	42.88 ± 6.05	40.82 ± 6.06
CD8 (% of CD45)					
Young placebo	13.42 ± 3.12	13.94 ± 2.32	13.56 ± 2.98	13.55 ± 1.33	14.64 ± 1.61
Old placebo	15.75 ± 7.37	15.87 ± 8.04	15.91 ± 7.64	15.84 ± 7.46	15.86 ± 8.62
Young HCQ	12.567 ± 2.743	13.61 ± 4.27	12.08 ± 3.65	12.50 ± 3.15	13.63 ± 2.27
Old HCQ	14.49 ± 7.12	14.69 ± 7.44	14.47 ± 7.04	16.16 ± 6.89	15.21 ± 7.23
Treg (% of CD4)					
Young placebo	6.046 ± 1.053	5.461 ± 2.124	5.888 ± 0.794	5.544 ± 2.096	5.734 ± 0.815
Old placebo	7.494 ± 3.206	7.725 ± 2.983	7.667 ± 2.468	7.530 ± 2.452	7.599 ± 2.684
Young HCQ	6.410 ± 1.476	6.611 ± 1.293	6.096 ± 1.052	5.958 ± 2.259	6.884 ± 0.852
Old HCQ	6.172 ± 1.561	6.160 ± 1.655	6.588 ± 1.842	6.340 ± 1.681	6.739 ± 1.870
NK (% of CD45)					
Young placebo	10.15 ± 6.05	10.91 ± 5.17	9.82 ± 4.69	9.51 ± 5.14	9.20 ± 4.51
Old placebo	13.78 ± 3.66	16.61 ± 3.34	15.00 ± 3.71	13.95 ± 3.00	14.44 ± 4.34
Young HCQ	8.15 ± 3.03	8.69 ± 3.56	7.48 ± 3.32	7.55 ± 3.10	8.25 ± 2.38
Old HCQ	13.02 ± 5.20	13.40 ± 5.79	13.26 ± 6.47	11.86 ± 5.70	11.93 ± 5.35
B cells (% of CD45)					
Young placebo	9.25 ± 2.90	9.82 ± 3.66	9.58 ± 3.46	9.03 ± 3.45	9.49 ± 2.89
Old placebo	9.13 ± 2.71	9.12 ± 2.64	10.26 ± 3.07	10.10 ± 3.07	9.96 ± 3.33
Young HCQ	7.36 ± 3.43	8.33 ± 3.48	8.27 ± 3.31	7.67 ± 3.49	8.29 ± 3.55
Old HCQ	11.45 ± 3.19	10.84 ± 3.82	11.21 ± 2.87	10.71 ± 2.70	11.26 ± 3.54
Breg ($CD5^+$ $CD1d^{hi}$)					
Young placebo	0.599 ± 0.236	0.759 ± 0.490	0.599 ± 0.219	0.625 ± 0.184	0.646 ± 0.160
Old placebo	0.720 ± 0.238	0.519 ± 0.189	0.973 ± 0.538	0.783 ± 0.194	0.972 ± 0.955
Young HCQ	0.748 ± 0.339	1.229 ± 1.230	1.065 ± 0.624	0.755 ± 0.245	0.934 ± 0.514
Old HCQ	0.565 ± 0.197	0.548 ± 0.225	0.654 ± 0.236	0.855 ± 0.429	0.879 ± 0.698
Transitional B cells					
Young placebo	4.044 ± 2.204	4.295 ± 2.368	5.113 ± 2.223	4.736 ± 2.041	4.810 ± 2.216
Old placebo	4.014 ± 1.502	4.025 ± 1.520	4.223 ± 1.542	4.152 ± 1.331	3.849 ± 1.270
Young HCQ	3.900 ± 2.184	4.114 ± 2.234	4.848 ± 2.486	4.571 ± 1.819	4.576 ± 1.993
Old HCQ	4.131 ± 1.534	4.120 ± 1.606	4.375 ± 1.598	4.276 ± 1.577	4.341 ± 1.669
ASC					
Young placebo	0.955 ± 0.659	0.789 ± 0.446	0.660 ± 0.713	0.751 ± 0.502	1.135 ± 0.684
Old placebo	2.155 ± 3.520	1.359 ± 1.451	2.785 ± 4.223	1.205 ± 0.731	1.472 ± 1.006
Young HCQ	1.161 ± 0.780	1.197 ± 0.908	1.276 ± 1.078	1.304 ± 0.757	1.431 ± 1.021
Old HCQ	0.701 ± 0.439	0.618 ± 0.514	1.376 ± 0.607	0.971 ± 0.255	1.036 ± 0.818
Classical monocyte (% of CD45)					
Young placebo	2.033 ± 1.778	2.063 ± 1.222	2.205 ± 1.604	1.885 ± 1.481	2.276 ± 1.790
Old placebo	1.690 ± 0.926	2.095 ± 0.952	1.963 ± 0.969	2.219 ± 1.852	2.476 ± 1.276
Young HCQ	1.439 ± 1.308	1.699 ± 1.002	1.209 ± 1.181	1.579 ± 1.057	1.765 ± 1.276
Old HCQ	1.022 ± 0.588	1.131 ± 0.349	1.256 ± 0.613	0.920 ± 1.530	1.511 ± 0.693

Intermediate monocyte (% of CD45)					
Young placebo	0.377 ± 0.294	0.523 ± 0.484	0.503 ± 0.325	0.267 ± 0.131	0.546 ± 0.471
Old placebo	1.023 ± 0.527	0.983 ± 0.507	1.054 ± 0.488	0.825 ± 0.438	0.714 ± 0.370
Young HCQ	0.308 ± 0.170	0.337 ± 0.222	0.326 ± 0.152	0.247 ± 0.072	0.285 ± 0.115
Old HCQ	0.438 ± 0.232	0.561 ± 0.254	0.632 ± 0.238	0.428 ± 0.310	0.637 ± 0.561
Non-classical monocyte (% of CD45)					
Young placebo	1.822 ± 1.230	2.056 ± 1.439	3.336 ± 2.895	2.438 ± 3.217	2.192 ± 2.719
Old placebo	2.268 ± 1.259	3.252 ± 1.584	4.631 ± 3.291	4.957 ± 2.896	4.544 ± 3.432
Young HCQ	1.813 ± 1.382	2.056 ± 1.585	2.433 ± 1.441	1.099 ± 0.909	1.258 ± 1.001
Old HCQ	2.658 ± 2.939	2.308 ± 1.492	2.707 ± 1.819	2.360 ± 1.281	2.469 ± 1.002
Plasmacytoid DC (% of CD45)					
Young placebo	0.416 ± 0.409	0.273 ± 0.162	0.266 ± 0.169	0.242 ± 0.142	0.254 ± 0.204
Old placebo	0.244 ± 0.169	0.200 ± 0.080	0.207 ± 0.123	0.206 ± 0.093	0.283 ± 0.138
Young HCQ	0.212 ± 0.099	0.199 ± 0.114	0.175 ± 0.122	0.183 ± 0.111	0.197 ± 0.125
Old HCQ	0.260 ± 0.612	0.175 ± 0.060	0.219 ± 0.091	0.201 ± 0.119	0.244 ± 0.067

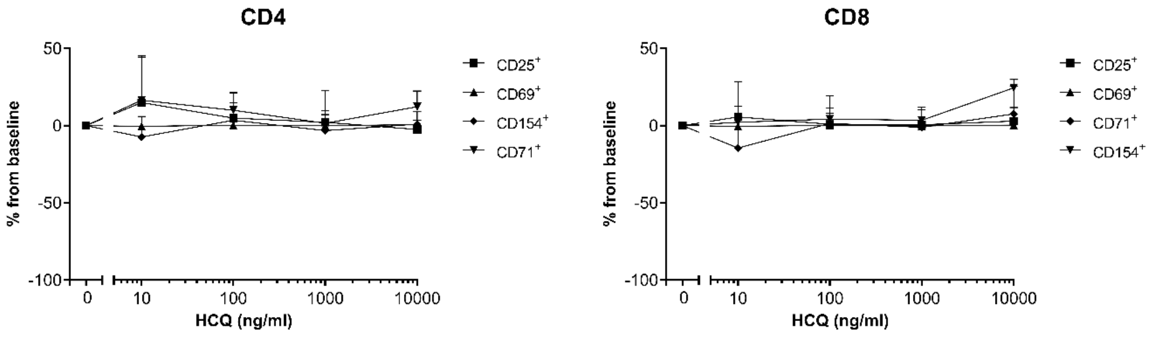

Figure S1: HCQ does not inhibit T cell activation in vitro

Activation markers CD25, CD69, CD154 and CD71 were measured by flow cytometry on $CD4^+$ T cells (left panel) and $CD8^+$ T cells (right panel), after whole blood stimulation with PHA for 6 hours in the presence of hydroxychloroquine (HCQ). Change from baseline is shown, mean and SD of 6 healthy donors.

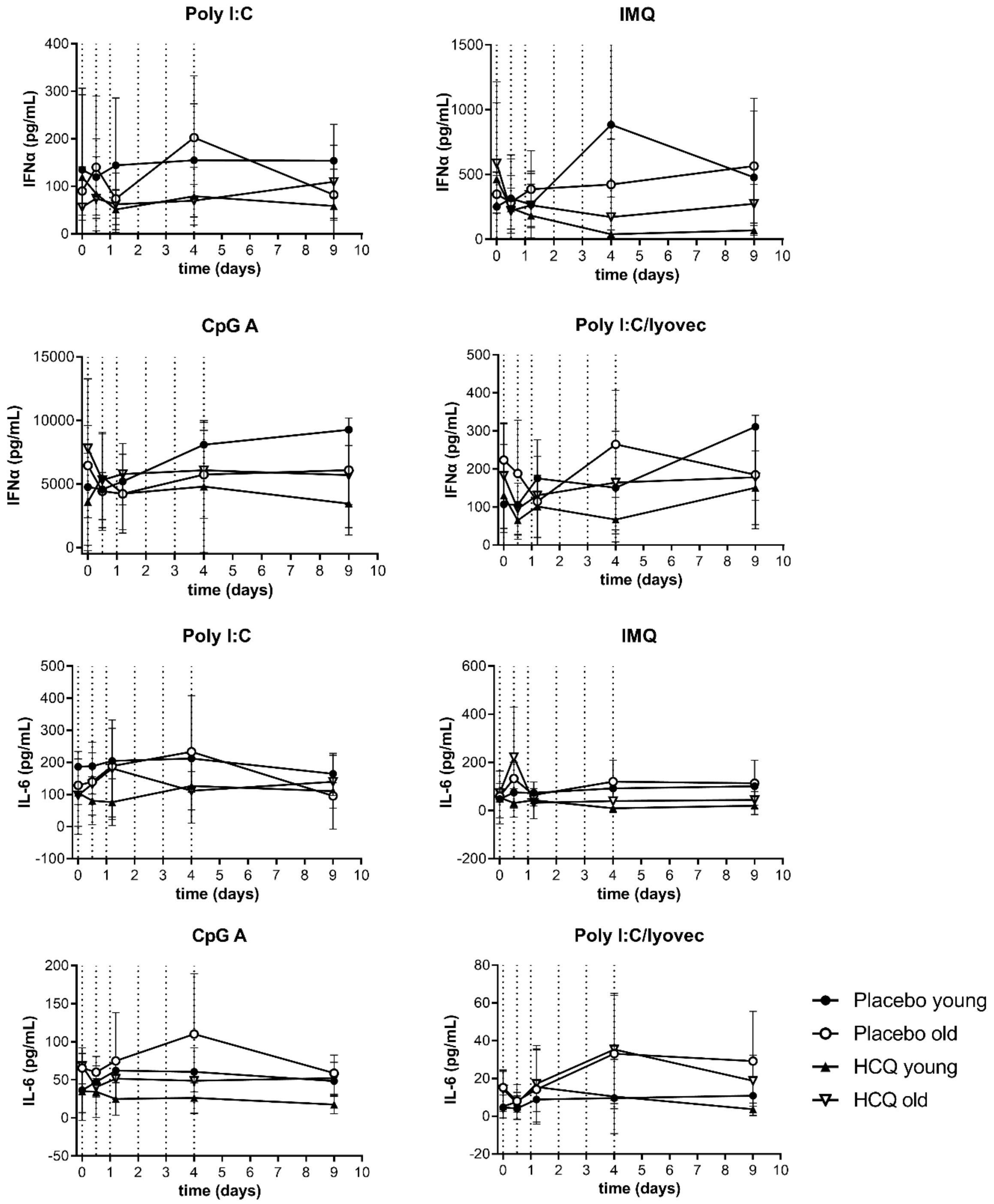

Figure S2: in vivo HCQ effects on IFNα and IL-6 after in vivo HCQ dosing per age and treatment group

Effects of hydroxychloroquine (HCQ) on IFNα release following stimulations with Poly I:C (TLR3), Imiquimod (TLR7), CpG A (TLR9) and poly I:C/lyovec (RIG-I) on isolated PBMCs. Data is shown as mean + SD as one-sided error bars. Dotted vertical lines indicate HCQ dosing times.

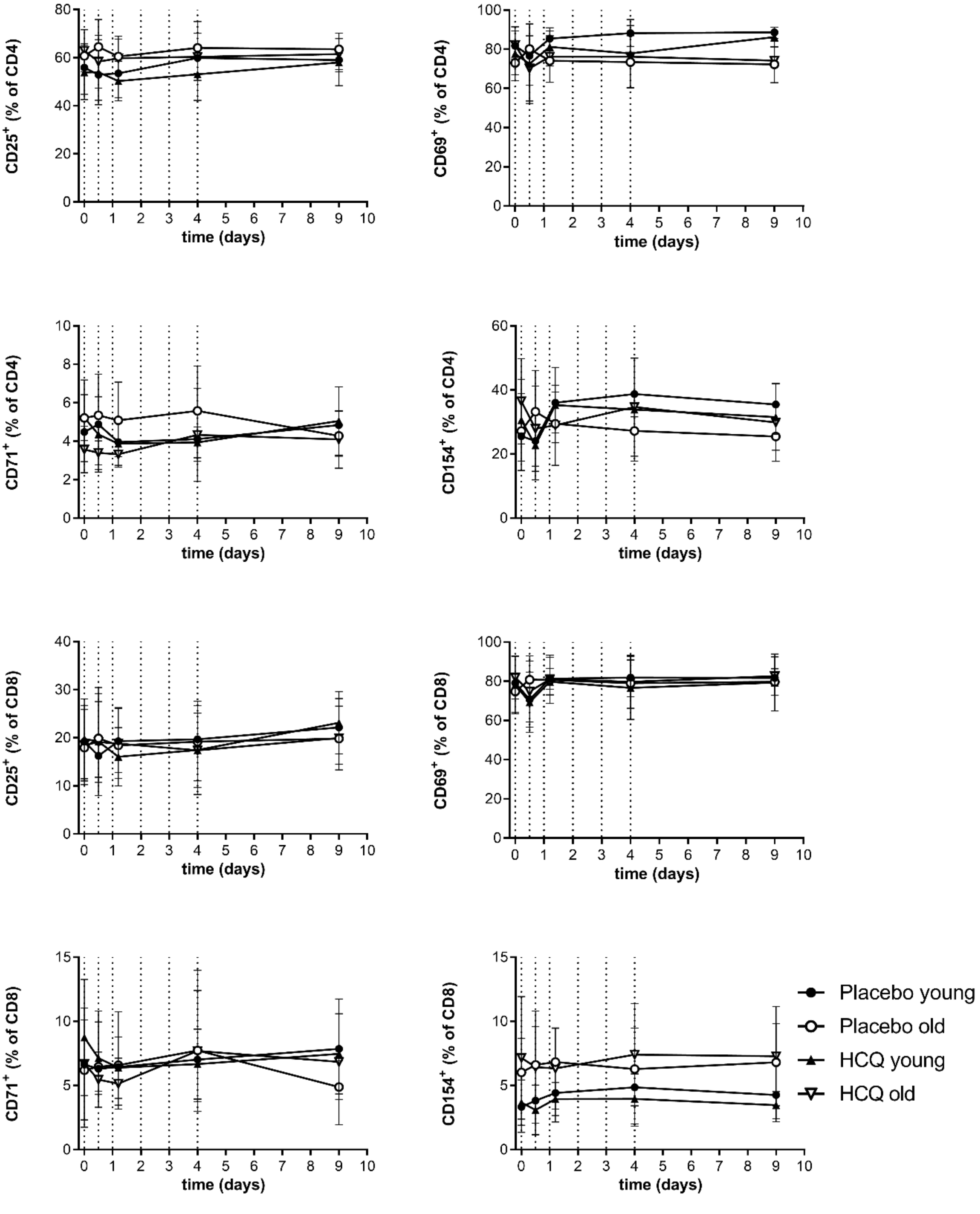

Figure S3: HCQ does not inhibit T cell activation in vivo

Activation markers CD25, CD69, CD154 and CD71 were measured by flow cytometry on $CD4^+$ T cells (left panel) and $CD8^+$ T cells (right panel), after whole blood stimulation with PHA for 6 hours. Data is shown as mean + SD. Dotted vertical lines indicate HCQ dosing times.

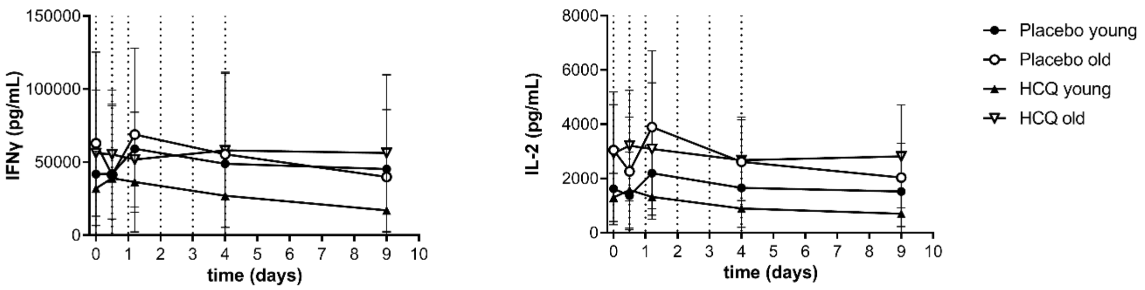

Figure S4: HCQ does not inhibit IFNγ and IL-2 release after ex vivo T cell stimulation

IFNγ (left) and IL-2 (right) release was measured by ELISA after whole blood incubation with PHA for 6 hours. The mean ± SD are shown of the treatment and age groups. Dotted vertical lines indicate HCQ dosing times.

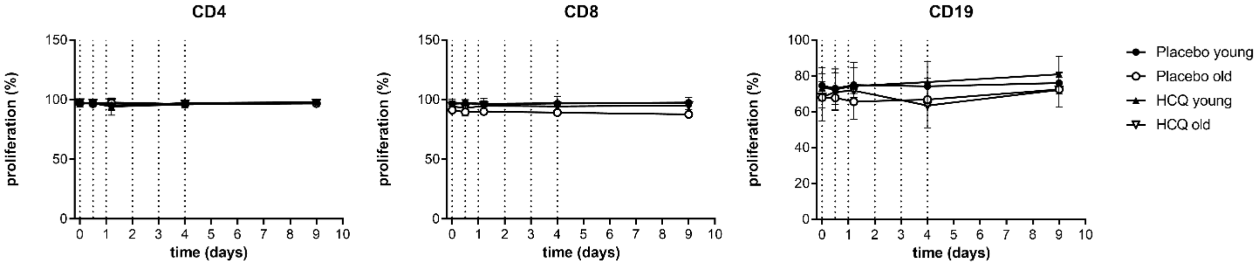

Figure S5: In vivo HCQ effect on T and B cell proliferation, split for age groups.

PBMCs were stained with CTV and stimulated for 5 days with 5µg/ml PHA for T cell proliferation (A), or 5µg/mL anti-CD40 mAb + 2.5 µM CpG B for B cell proliferation (B). Proliferation was measured by flow cytometry. The mean ± SD are shown of the treatment and age groups. Dotted vertical lines indicate HCQ dosing times.
